# Loss of AtPLC1 Impairs Salt–Alkali Tolerance via Disruption of Stomatal Regulation and Redox Homeostasis in *Arabidopsis thaliana*

**DOI:** 10.3390/plants15172633

**Published:** 2026-08-28

**Authors:** Xiang Li, Yu Wang, Linhan Si, Daqian Sun, Nan Wang, Weican Liu, Yuanyuan Dong, Xiaowei Li, Fawei Wang

**Affiliations:** College of Life Science, Jilin Agricultural University, Changchun 130118, China; lx15061765287@163.com (X.L.); wy1967097@163.com (Y.W.); 15090281978@163.com (L.S.); dqsun1993@163.com (D.S.); wangnanlunwen@126.com (N.W.); liuweican602@163.com (W.L.); yydong23@163.com (Y.D.); xiaoweili1206@163.com (X.L.)

**Keywords:** *Arabidopsis thaliana*, salt–alkali tolerance, *AtPLC1*, ion homeostasis, stomatal regulation, oxidative stress response

## Abstract

Soil salinization poses a major environmental threat to global agriculture, affecting approximately 20% of cultivated land and 50% of irrigated land worldwide. Developing salt–alkali tolerant plant varieties represents a sustainable strategy for utilizing these marginal lands. Phosphatidylinositol-specific phospholipase C (PI-PLC) is a key enzyme in the phosphoinositide signaling system and has been implicated in plant stress responses; however, its function under salt–alkali stress remains poorly understood. In this study, the function of *AtPLC1* in salt–alkali tolerance was investigated, and only the *atplc1* mutant exhibited a pronounced stress-sensitive phenotype, with *AtPLC1* being predominantly expressed in roots and leaves, with peak expression at 6 h of treatment. Compared with wild-type, *atplc1* mutants displayed significantly reduced seedling survival, retarded root growth, decreased biomass, water content, chlorophyll, and soluble sugar contents, yet accumulated higher levels of Na^+^, malondialdehyde, H_2_O_2_, and superoxide anions under salt–alkali stress. Notably, *atplc1* mutants showed increased stomatal conductance and decreased leaf surface temperature, as detected by thermal imaging, indicating impaired water regulation. Collectively, our findings demonstrate that *AtPLC1* positively regulates salt–alkali tolerance and provides a candidate gene for molecular breeding of stress-resistant crops.

## 1. Introduction

Salt stress and alkali stress are major abiotic constraints that profoundly affect plant growth and development, posing a serious threat to global agricultural productivity [1]. Currently, approximately 950 million hectares of land worldwide are affected by salinization, and the extent of salinized areas is projected to expand further, with estimates suggesting that up to 50% of global cultivated land could be lost by 2050 [2,3]. The damage inflicted by salt and alkali stresses in salt–alkali soils can retard plant growth or even lead to plant death. Therefore, elucidating the mechanisms underlying plant tolerance to salt–alkali stress and employing molecular biotechnology to engineer stress-tolerant plants have become research priorities in plant and agricultural sciences [4,5].

Phospholipase C (PLC) is a family of hydrolases that plays critical roles in plant signal transduction and lipid metabolism. Based on their substrate specificities, plant PLCs are mainly classified into two major groups: phosphatidylinositol-specific PLC (PI-PLC) and non-specific PLC (NPC). PI-PLC specifically hydrolyzes phosphatidylinositol 4,5-bisphosphate (PIP_2_) to generate inositol 1,4,5-trisphosphate (IP_3_) and diacylglycerol (DAG) [6]. Unlike in animals, plants lack IP_3_ receptor-mediated Ca^2+^ release from intracellular stores; however, IP_3_ and its phosphorylated derivatives (e.g., IP_6_) can still modulate Ca^2+^ signaling by regulating ion channel activities. DAG, on the other hand, is considered a lipid signaling molecule in plants, potentially involved in membrane lipid remodeling or serving as a substrate for further metabolism into phosphatidic acid (PA), thereby activating downstream signaling cascades [7,8]. Given the central position of PLC in generating these lipid-derived signals, its physiological functions in plant growth, development, and stress adaptation have attracted considerable research interest.

In recent years, PLC family members have been cloned and functionally analyzed in various crop plants, such as rice, maize, soybean, tomato, and sorghum, highlighting their conserved and divergent roles across species [9,10,11,12,13]. PI-PLCs are involved in regulating diverse physiological processes, including pollen tube growth [14], stomatal movement [15], seed germination [16], root development [17], and adaptive responses to environmental stresses [18,19]. AtPLC1 is a prominent member of the phosphatidylinositol-specific phospholipase C (PI-PLC) family in *A. thaliana*. Previous studies have demonstrated that AtPLC1 participates in the regulation of multiple biological processes, including trichome development, flavonoid pigment biosynthesis, and differentiation of mucilage-producing cells of the seed coat, likely through its role in phosphoinositide signaling and genetic interaction with the transcription factor TTG2 [20]. In the context of drought stress responses, AtPLC1 has been shown to function in the abscisic acid (ABA) signaling pathway—its elevated activity and the consequent increase in IP_3_ levels are necessary for maximal ABA-induced gene expression; however, overexpression of AtPLC1 alone is not sufficient to trigger the expression of ABA-responsive genes, which suggests that AtPLC1 primarily participates in secondary ABA responses rather than direct signal perception [21]. Furthermore, AtPLC1 has been implicated in plant growth, development, and adaptation to drought stress [22]. Nevertheless, whether AtPLC1 is involved in the regulation of salt–alkali stress tolerance remains largely unknown.

Plant tolerance to salt–alkali stress is a complex quantitative trait involving coordinated regulation of multiple genes and signaling pathways [23]. The currently recognized core mechanisms primarily revolve around three major aspects: ion homeostasis remodeling, osmotic adjustment, and reactive oxygen species (ROS) scavenging [24,25,26]. However, whether and how PLC-mediated lipid signaling contributes to these adaptive mechanisms under salt–alkali stress remains unclear. In this study, we employed *A. thaliana atplc1* mutants to systematically analyze the potential function of AtPLC1 in salt–alkali stress responses, as this gene was found to be transcriptionally induced by salt–alkali stress and its loss-of-function mutants exhibited pronounced stress hypersensitivity. Our aim was to elucidate the molecular mechanisms by which PLC-mediated lipid signaling contributes to plant salt–alkali tolerance.

## 2. Results

### 2.1. Expression Patterns of the PLC Gene Family in A. thaliana

**Figure 1:**
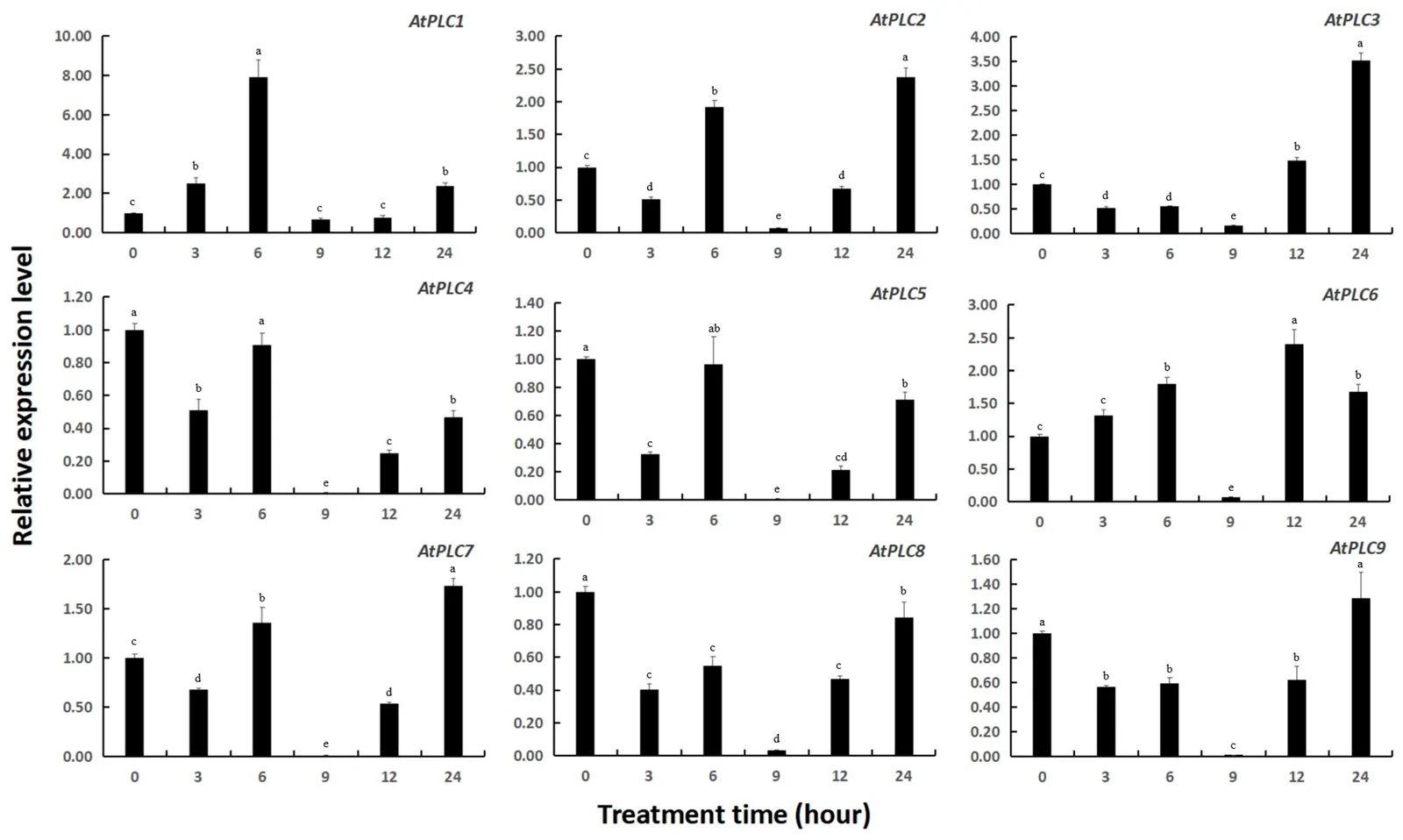
Expression analysis of the PLC gene family in *A. thaliana*. Different lowercase letters indicate significant differences according to Tukey’s multiple comparison test (*p* < 0.05).

To investigate the potential involvement of the PLC gene family in response to salt–alkali stress, we first examined their transcriptional profiles in leaves of six-leaf-stage *A. thaliana* seedlings subjected to stress treatment for 0, 3, 6, 9, 12, and 24 h using qRT-PCR. The expression of *AtPLC1* peaked at 6 h after stress initiation, suggesting an early and transient response. In contrast, the transcript levels of *AtPLC2*, *AtPLC3*, and *AtPLC9* exhibited a continuous increase over the entire treatment period, with the highest expression observed at 24 h. Interestingly, a gradual decrease in expression was detected for *AtPLC4*, *AtPLC5*, and *AtPLC8* as the stress duration prolonged. Furthermore, *AtPLC6* showed a distinct expression pattern, reaching its maximum level at 12 h post-treatment (Figure 1). These results indicate that members of the PLC gene family display diverse and time-dependent expression dynamics in response to salt–alkali stress in *A. thaliana* leaves.

### 2.2. Phenotypic Characterization of Atplcs Mutants Under Salt–Alkali Stress

**Figure 2:**
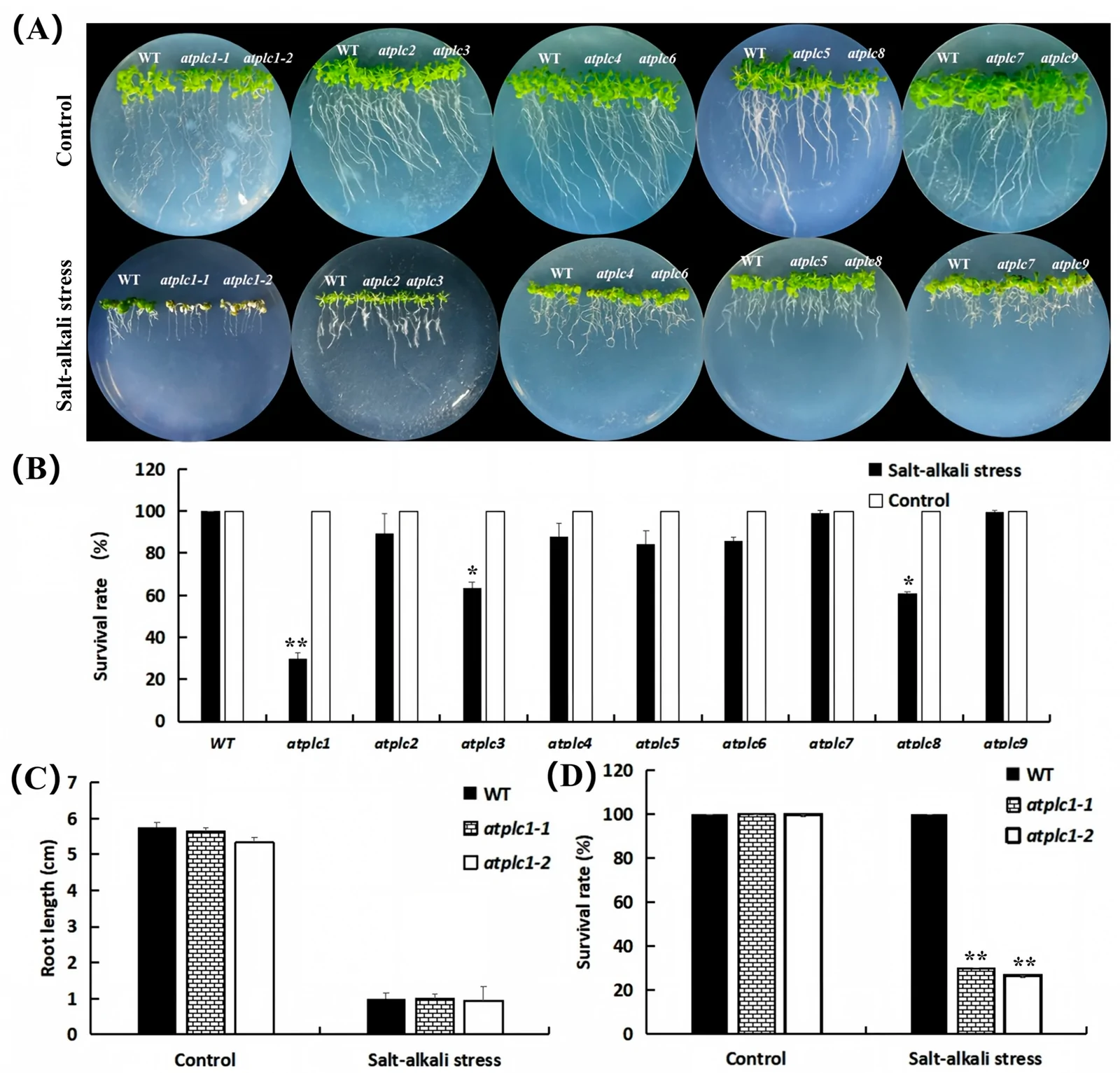
Root length and survival rate of different *atplcs* mutants under salt–alkali stress. (**A**) Phenotypes of WT and *atplcs* mutants under salt–alkali stress. (**B**) Survival rates of WT and *atplcs* mutants after salt–alkali treatment. (**C**) Root length of WT and *atplc1* mutants under salt–alkali stress. (**D**) Survival rates of WT and *atplc1* mutants after salt–alkali treatment. Values are means ± SD (n = 3, * *p* < 0.05, ** *p* < 0.01, *t*-test).

To investigate whether the PLC gene family in *A. thaliana* is involved in regulating plant tolerance to salt–alkali stress, we first purchased and identified homozygous *atplcs* mutants (Figure S1). Wild-type *A. thaliana* and *atplcs* mutants with different T-DNA insertion sites were subjected to salt–alkali stress treatment. Under control conditions, no significant differences in survival rates were observed between WT and all *atplcs* mutants. However, under salt–alkali stress, the survival rates of *atplc1*, *atplc3*, and *atplc8* mutants were significantly lower than those of other lines, with the *atplc1* mutant exhibiting a survival rate of only 28.35% (Figure 2A,B). This suggests that *AtPLC1* may play a critical regulatory role in the salt–alkali tolerance of *A. thaliana*. To further confirm this role, we analyzed the root length and survival rate of two *AtPLC1* mutants, *atplc1-1* and *atplc1-2*, under salt–alkali stress. The results showed that although root development in both WT and *atplc1-1* and *atplc1-2* was severely inhibited after stress, no significant differences were observed among them. However, the survival rates of *atplc1-1* and *atplc1-2* were significantly lower than those of WT plants (Figure 2C,D). These results indicate that loss of *AtPLC1* reduces the tolerance of *A. thaliana* to salt–alkali stress.

### 2.3. AtPLC1 Mediates Osmotic Adjustment and Membrane Integrity Under Salt–Alkali Stress

**Figure 3:**
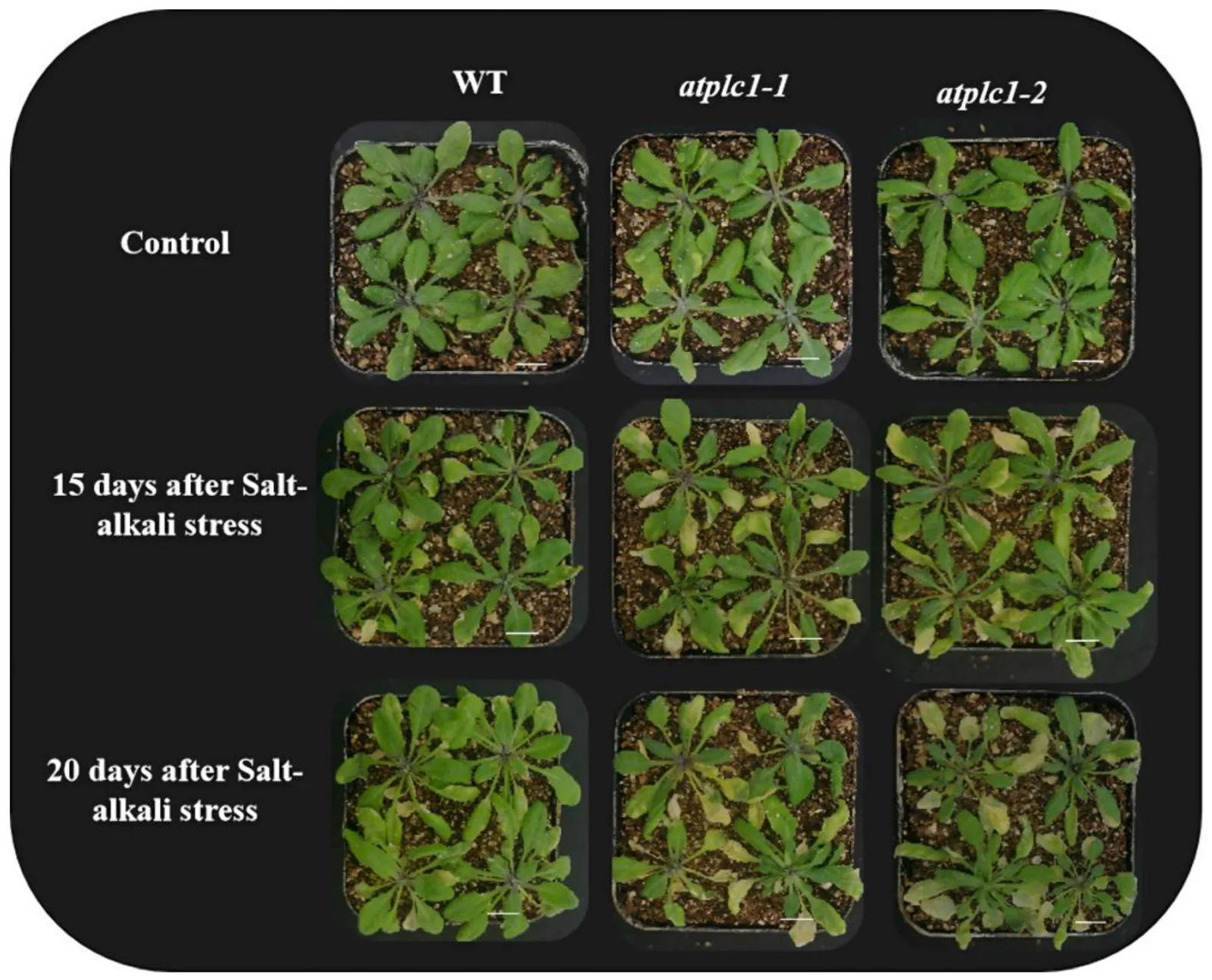
Phenotypic analysis of the *A. thaliana atplc1* mutants under salt–alkali stress. Scale bars = 1 cm.

**Figure 4:**
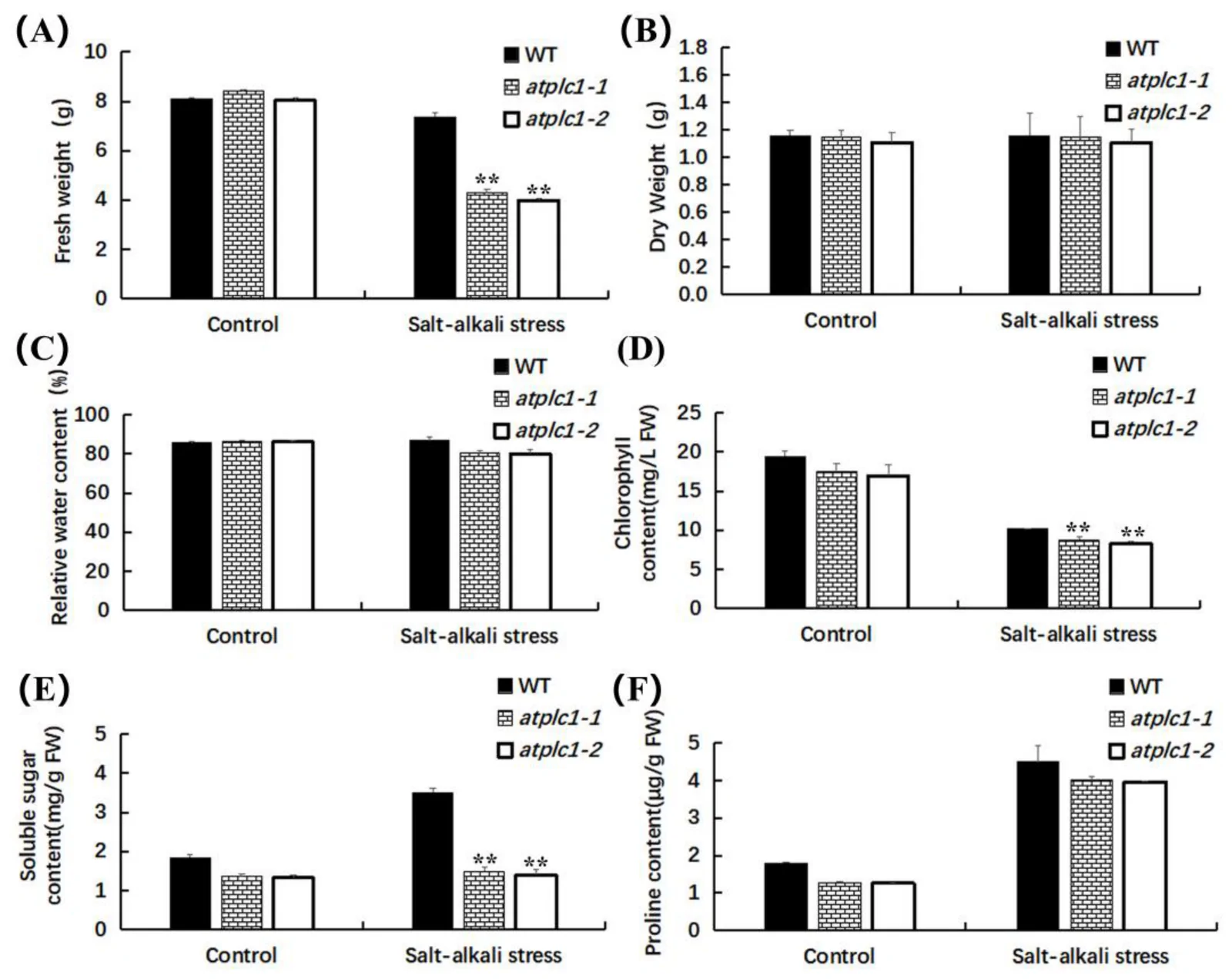
Physiological and biochemical parameters of the *A. thaliana atplc1* mutants under salt–alkali stress. Measurements of (**A**) fresh weight, (**B**) dry weight, (**C**) relative water content, (**D**) chlorophyll content, (**E**) soluble sugar content, and (**F**) proline content. Values are means ± SD (n = 3, ** *p* < 0.01, *t*-test).

To further confirm the regulatory role of *AtPLC1* under salt–alkali stress, WT, *atplc1-1*, and *atplc1-2* were subjected to salt–alkali stress treatment in soil. The results showed that after stress, *atplc1-1* and *atplc1-2* exhibited varying degrees of leaf wilting and chlorosis (Figure 3). Measurement of fresh weight, dry weight, and water content before and after stress revealed that salt–alkali stress only affected fresh weight, while dry weight remained unchanged. Although the relative water content of *atplc1* mutants was lower than that of WT after stress, the difference was not statistically significant (Figure 4A–C). To further investigate this regulatory effect, we analyzed chlorophyll content, soluble sugar, and proline content. After salt–alkali stress, chlorophyll and soluble sugar contents in *atplc1* mutants were significantly lower than those in WT, whereas no significant difference was observed in proline content (Figure 4D–F).

**Figure 5:**
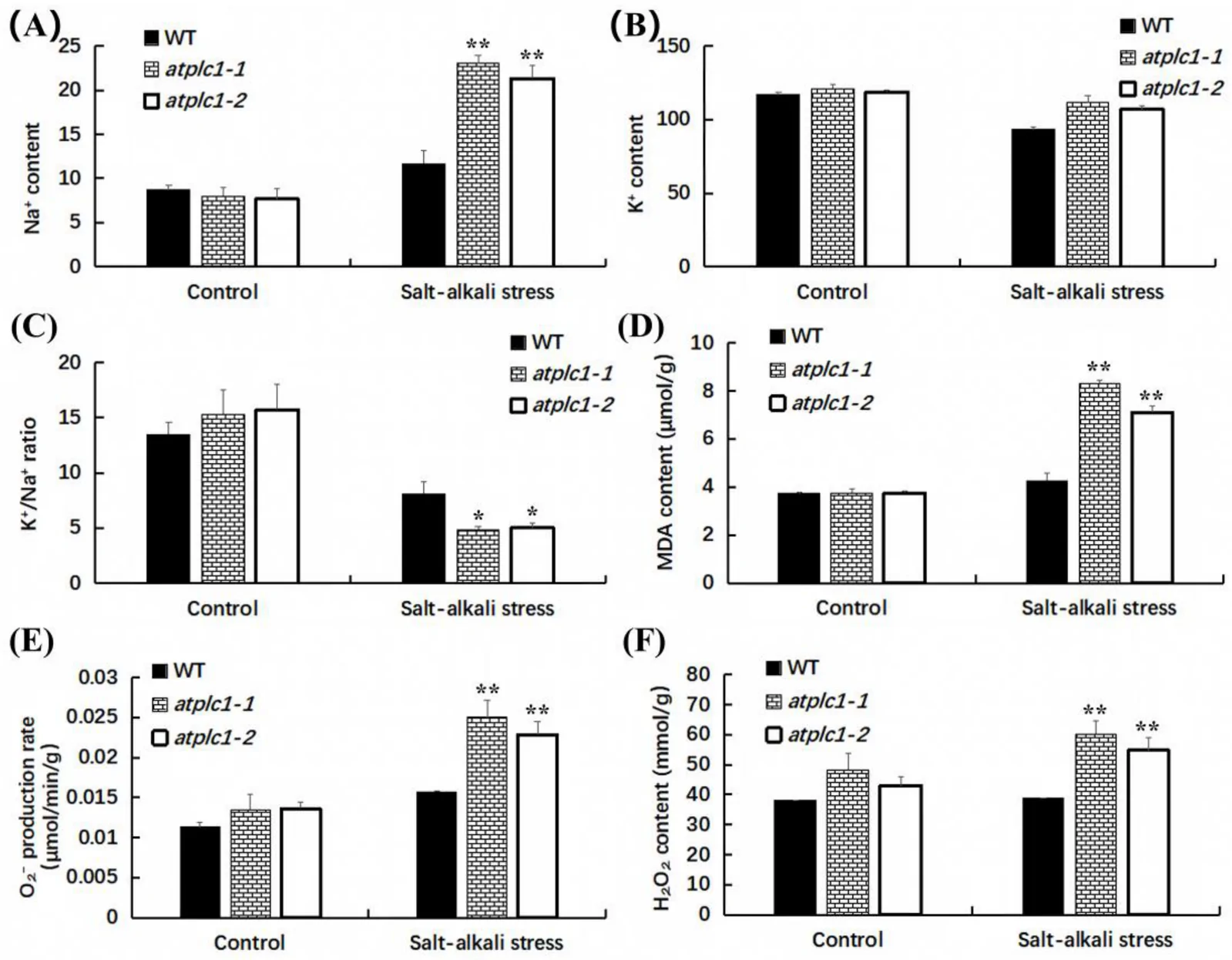
Ionic and oxidative stress responses under salt–alkali stress. Measurements of (**A**) Na^+^ content, (**B**) K^+^ content, (**C**) K^+^/Na^+^ ratio, (**D**) MDA content, (**E**) O_2_^−^ production rate, and (**F**) H_2_O_2_ content. Values are means ± SD (n = 4, * *p* < 0.05, ** *p* < 0.01, *t*-test).

To elucidate how *AtPLC1* participates in the *A. thaliana* response to salt–alkali stress, we analyzed sodium and potassium ion contents as well as oxidative stress-related indicators. The results showed that after salt–alkali stress, both Na^+^ and K^+^ contents were significantly elevated in *atplc1* mutants, while the K^+^/Na^+^ ratio was significantly reduced (Figure 5A–C), suggesting that *AtPLC1* may regulate salt–alkali tolerance through modulation of sodium and potassium ion homeostasis. Measurements of MDA, superoxide anion, and hydrogen peroxide contents revealed that oxidative stress-related indicators in *atplc1* mutants were significantly higher than those in WT (Figure 5D–F), indicating that this gene may participate in the salt–alkali stress response through regulation of reactive oxygen species (ROS).

### 2.4. AtPLC1 Modulates the Antioxidant System to Alleviate Oxidative Damage

**Figure 6:**
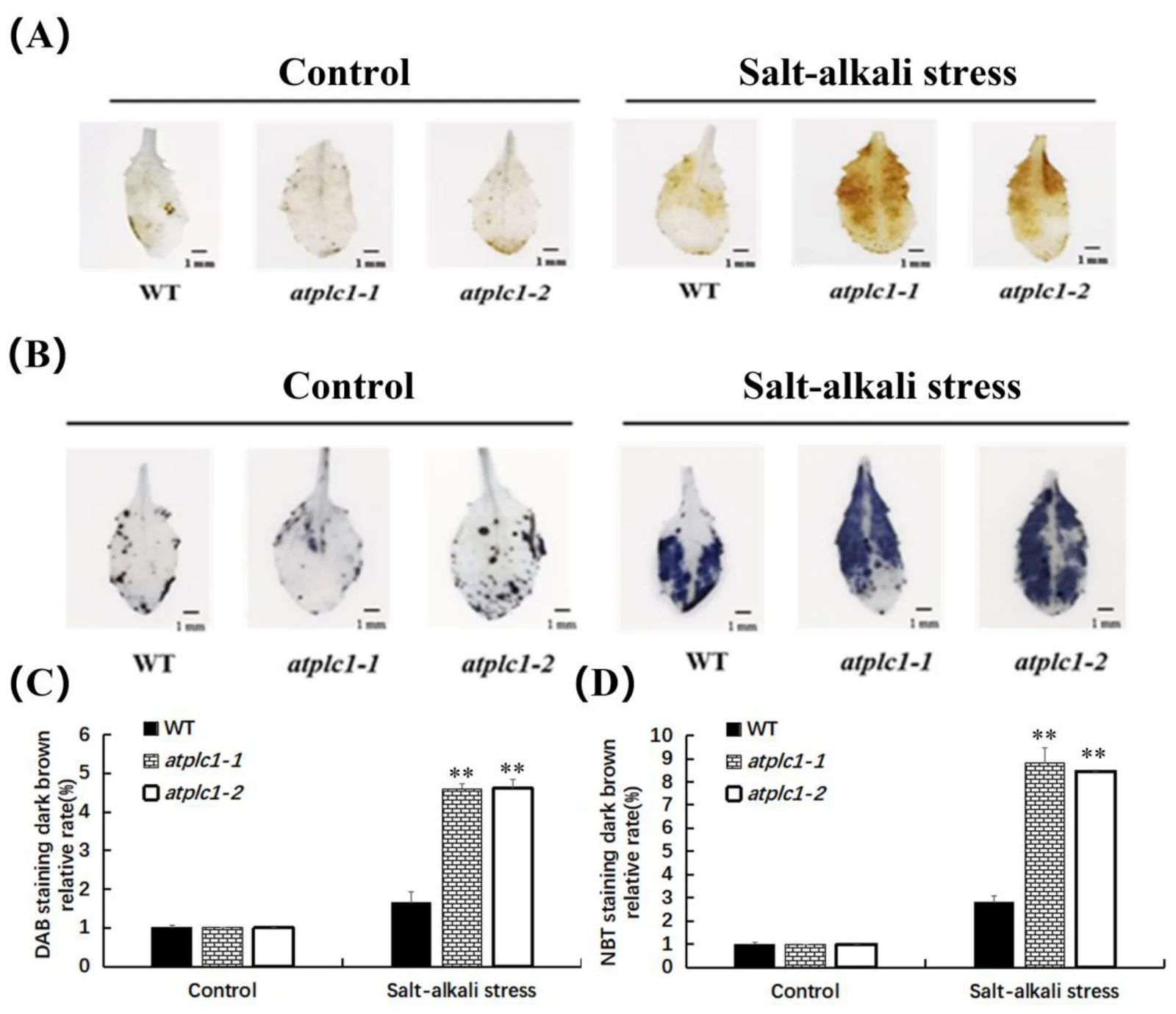
Histochemical detection and quantitative analysis of reactive oxygen species accumulation. (**A**) DAB staining for H_2_O_2_ detection. (**B**) NBT staining for O_2_^−^ detection. (**C**) Relative DAB staining intensity. (**D**) Relative NBT staining intensity (n = 37, ** *p* < 0.01, *t*-test).

To further confirm how *AtPLC1* regulates ROS levels in *A. thaliana* under salt–alkali stress, we performed DAB and NBT staining analyses on leaves of WT and *atplc1* mutants subjected to salt–alkali stress. In plant cells, peroxidase reacts with H_2_O_2_ to form yellow precipitates, reflecting the oxidative stress status in vivo. DAB staining revealed that no obvious yellow precipitates were detected in any untreated lines. After salt–alkali stress, WT plants exhibited slight yellow precipitates, whereas *atplc1* mutant leaves displayed extensive yellow precipitates, indicating that mutant plants generated large amounts of H_2_O_2_ following stress (Figure 6A,C). NBT staining was used to assess O_2_^−^ levels in plants. Untreated lines showed few blue spots, while after stress, WT exhibited some blue spots, and *atplc1* mutant leaves showed abundant blue spots. Quantitative analysis revealed that the NBT-stained areas in *atplc1* mutants were 3.04- to 3.16-fold higher than those in WT after stress (Figure 6B,D). These results demonstrate that loss of *AtPLC1* function significantly exacerbates the accumulation of H_2_O_2_ and O_2_^−^ in *A. thaliana* leaves under salt–alkali stress, indicating that *AtPLC1* negatively regulates ROS burst in the stress response, and its deficiency leads to more severe oxidative damage.

### 2.5. PLC Regulates Stomatal Movement and Leaf Thermal Balance Under Stress

**Figure 7:**
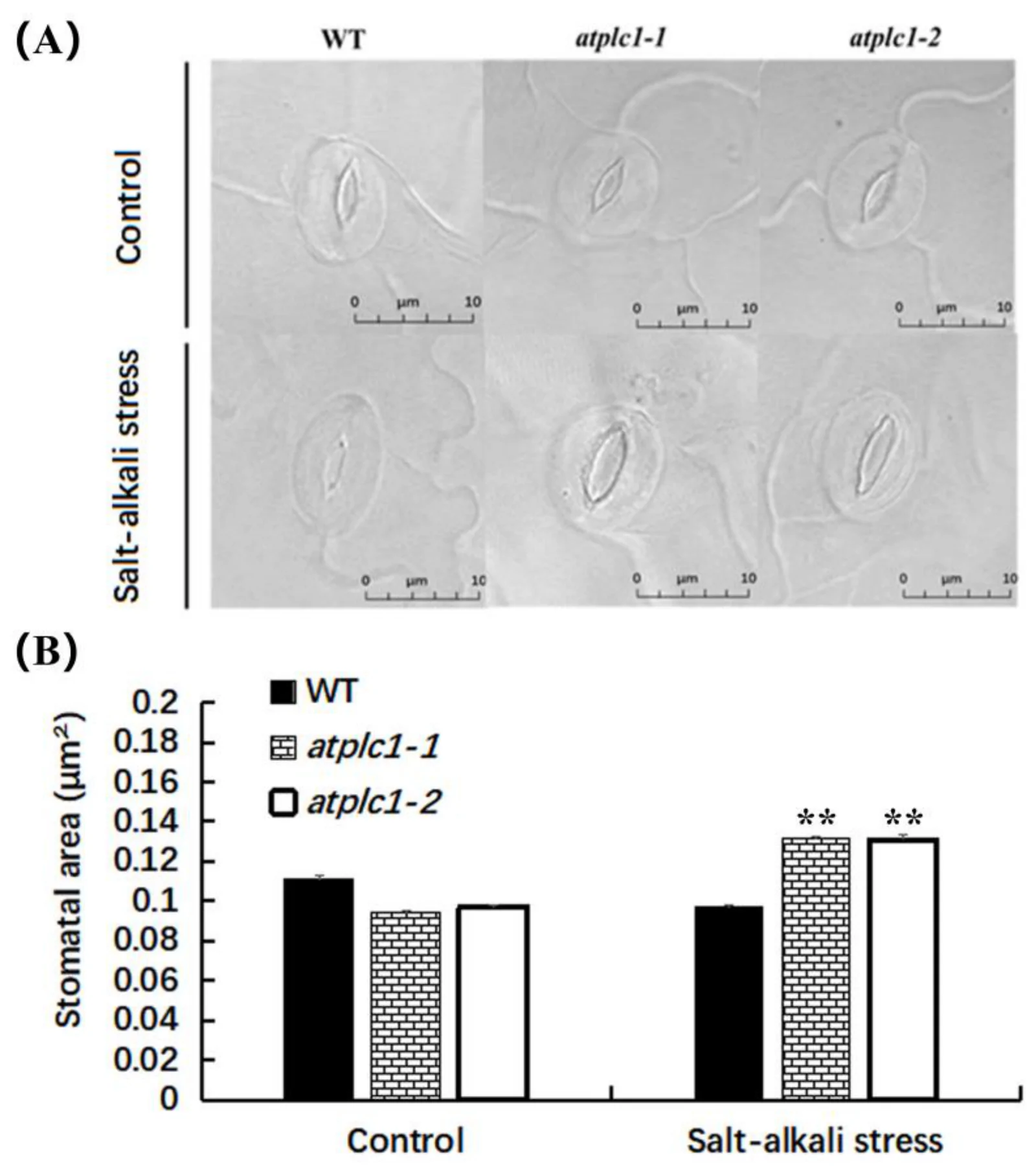
Stomatal morphological changes and stomatal area quantification in leaves under salt–alkali stress. (**A**) Representative microscopic images of stomata. (**B**) Quantification of stomatal area (n = 83, ** *p* < 0.01, *t*-test).

**Figure 8:**
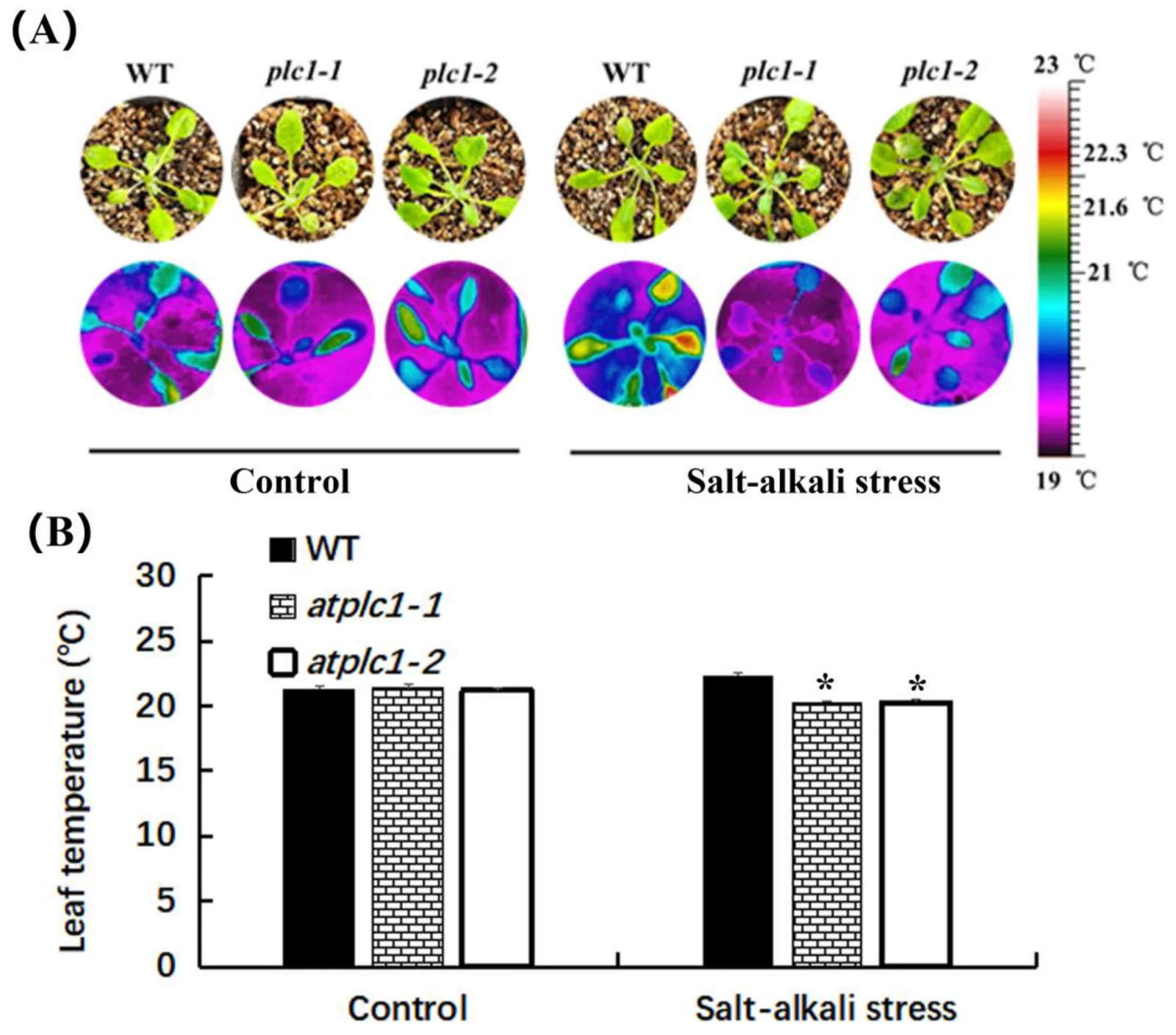
Leaf surface temperature analysis under salt–alkali stress. (**A**) Representative infrared thermographic images of leaves. (**B**) Quantification of leaf surface temperature (n = 46, * *p* < 0.05, *t*-test).

Based on the above ROS differences, we further analyzed stomatal movement and surface temperature changes in WT and *atplc1* mutant leaves under salt–alkali stress through stomatal observation and infrared thermography, aiming to evaluate the impact of *AtPLC1* deficiency on transpirational cooling. Under non-stressed conditions, stomatal area in WT leaves was larger than that in *atplc1* mutants, but the difference was not significant. However, after salt–alkali stress, the stomatal area in *atplc1* mutants was significantly larger than that in WT, with an increase of 1.35-fold (Figure 7A,B). This indicates that under salt–alkali stress, loss of *AtPLC1* impairs stomatal closure, preventing mutant stomata from effectively responding to stress signals and contracting, which may compromise transpiration regulation and exacerbate water loss and physiological imbalance. Given that stomatal closure is impaired in the mutant under stress, its transpirational cooling capacity is likely altered, prompting us to further monitor leaf temperature changes via infrared thermography. The results showed that leaf temperatures of all lines were similar under control conditions, whereas after salt–alkali stress, *atplc1* mutant leaves exhibited significantly lower temperatures than WT plants (Figure 8A,B). Collectively, these results demonstrate that *AtPLC1* deficiency impairs stress-induced stomatal closure, leading to excessive transpirational water loss and enhanced leaf cooling, which together reflect a compromised ability to cope with salt–alkali stress.

## 3. Discussion

In *A. thaliana*, the PLC gene family comprises nine members (*AtPLC1*-*AtPLC9*), which exhibit diverse expression patterns in response to various abiotic stresses. In this study, expression dynamics of *AtPLC* family members upon salt–alkali stress treatment revealed that *AtPLC1* was rapidly induced at the early stage (6 h), whereas *AtPLC2*, *AtPLC3*, and *AtPLC9* showed continuously elevated expression throughout the treatment period. This early induction of *AtPLC1* is consistent with previous findings that *AtPLC1* transcript levels are significantly upregulated by dehydration and salt stress [27,28]. Notably, it has also been shown that *AtPLC1* expression returns to basal levels after prolonged stress exposure, which aligns with our observation that *AtPLC1* induction was transient rather than sustained [27]. Together, these findings demonstrate that *AtPLC1* serves as an early stress-responsive gene, whereas other PLC members may contribute to sustained adaptive responses.

Our phenotypic analysis revealed that *AtPLC1* is required for the survival of *A. thaliana* seedlings under salt–alkali stress, as the survival rate of *atplc1* mutants was drastically reduced to only 28.35% compared with wild-type plants. This observation is consistent with previous reports that PI-PLCs play protective roles under various stress conditions [9,29]. Notably, although the overall survival of *atplc1* mutants was severely compromised, their root growth inhibition under stress did not differ significantly from that of WT, suggesting that *AtPLC1* primarily affects post-germination viability and whole-plant stress tolerance rather than root elongation. This root-insensitive phenotype is reminiscent of findings in other stress-signaling mutants, where survival and root growth appear to be governed by partially independent genetic pathways [30,31]. The more pronounced effect on survival than on root growth implies that *AtPLC1* may be more critically involved in systemic stress adaptation, possibly through its roles in ion homeostasis, ROS signaling, or stomatal regulation, rather than locally mediating root developmental plasticity. Collectively, these results establish that *AtPLC1* is a positive regulator of salt–alkali tolerance, with its deficiency rendering plants hypersensitive to stress at the whole-plant level without necessarily affecting root growth dynamics.

In addition to the phenotypic observations, physiological assessments revealed that salt–alkali stress significantly reduced the fresh weight of both WT and *atplc1* mutants, whereas dry weight remained unchanged, indicating that the observed growth suppression was primarily due to water loss rather than a reduction in biomass accumulation. This is consistent with the notion that salt–alkali stress imposes an immediate osmotic constraint on plant cells without necessarily affecting the structural dry matter [32]. Although the relative water content of *atplc1* mutants was lower than that of WT under stress, the difference did not reach statistical significance, suggesting that *AtPLC1* deficiency does not drastically impair bulk tissue water retention under the tested conditions. However, the significantly reduced chlorophyll content in *atplc1* mutants points to accelerated chlorophyll degradation or impaired biosynthesis under stress, which likely contributes to the observed leaf chlorosis and reduced photosynthetic capacity [33,34,35]. In addition, the marked decrease in soluble sugar content in *atplc1* mutants implies that *AtPLC1* may be required for the accumulation of compatible solutes, which are crucial for osmotic adjustment and cellular protection under salt–alkali stress [36]. Interestingly, proline content did not differ significantly between WT and mutants, suggesting that proline accumulation may not be the primary osmotic adjustment strategy dependent on *AtPLC1*, or that other osmoprotectants (e.g., glycine betaine or trehalose) might compensate for this deficiency [37,38]. Collectively, these results indicate that *AtPLC1* contributes to salt–alkali tolerance by modulating chlorophyll stability and soluble sugar accumulation, whereas its impact on overall tissue water status appears limited under the present experimental conditions.

The substantial ion imbalance observed in *atplc1* mutants under salt–alkali stress further supports the involvement of *AtPLC1* in regulating ionic homeostasis. Compared with WT, *atplc1* mutants exhibited significantly elevated Na^+^ and K^+^ contents, leading to a markedly reduced K^+^/Na^+^ ratio—a well-established indicator of salt sensitivity and metabolic dysfunction [39,40,41,42]. Our results showed that both Na^+^ and K^+^ contents were significantly elevated in *atplc1* mutants, leading to a reduced K^+^/Na^+^ ratio. Although similar cases of concurrent Na^+^ and K^+^ elevation have been reported under certain stress conditions [43], this pattern deviates from the conventional model of salt-induced K^+^ depletion. The elevated K^+^ levels in *atplc1* mutants may reflect a disrupted regulatory feedback in which the mutant fails to coordinate ion transport properly, resulting in simultaneous accumulation of both cations. Collectively, these findings suggest that *AtPLC1* is essential for maintaining ionic homeostasis under salt–alkali stress, and its loss leads to a distinct ion dysregulation phenotype. In parallel, *atplc1* mutants displayed significantly higher levels of MDA, superoxide anion, and H_2_O_2_, indicating more severe oxidative damage. Similar increases in MDA and ROS levels have been observed in PI-PLC-deficient plants under stress conditions [44,45]. Collectively, these findings suggest that *AtPLC1* contributes to salt–alkali tolerance through coordinated regulation of ion homeostasis and oxidative stress defense, and its loss predisposes plants to both ionic toxicity and oxidative injury, ultimately compromising survival under stress.

Stomata in plant leaves serve as the gateway for gas exchange between the plant and the environment, and also represent the primary route for water loss. Our results demonstrate that loss of *AtPLC1* impairs stomatal closure under salt–alkali stress, leading to excessive water loss and reduced tolerance of *A. thaliana* to salt–alkali stress. This is consistent with previous findings that PI-PLC activity is closely linked to ABA-induced stomatal regulation and that disruption of PLC-mediated signaling compromises stress-induced stomatal closure [46,47,48]. These lines of evidence support the notion that *AtPLC1* plays a conserved and critical role in regulating stomatal movement under adverse conditions.

The distinct leaf temperature phenotype observed in *atplc1* mutants under salt–alkali stress is consistent with their impaired stomatal closure. As water evaporation from the leaf surface dissipates latent heat, the sustained transpirational water loss in mutants would inevitably result in enhanced evaporative cooling [49,50]. Similar thermal patterns have been reported in plants with constitutively open stomata or defective closure mechanisms, which tend to exhibit cooler leaf surfaces under high evaporative demand [51]. Although enhanced cooling may appear beneficial, it comes at the cost of excessive water loss, which exacerbates dehydration under salt–alkali stress. This trade-off highlights the importance of *AtPLC1*-mediated stomatal closure as an adaptive response. Collectively, our findings suggest that *AtPLC1* coordinates stomatal movement and thermal balance, and its deficiency prioritizes evaporative cooling over water retention, compromising plant survival under salt–alkali stress.

## 4. Materials and Methods

### 4.1. Plant Material and Treatment

*A. thaliana* seeds were sown on 1/2 MS medium and incubated in a growth chamber for 4 days prior to stress treatment (22 °C, light intensity of 6000–8000 lux, 12 h/12 h photoperiod, relative humidity of 30%). The stress medium consisted of 1/2 MS medium supplemented with a salt–alkali stress solution (140 mM NaCl + 10 mM NaHCO_3_). The seedlings were then returned to the growth chamber and cultured for an additional 15 days. Subsequently, root length was measured, and the survival rate of each line was calculated. Each treatment included four replicates.

Seeds of each *A. thaliana* line were uniformly sown in culture pots, with approximately 20 seeds per pot. During seedling growth, seedlings with inconsistent growth vigor were removed, leaving four seedlings per pot for subsequent stress treatment. For stress treatment, the pots were irrigated with a salt–alkali solution containing 140 mM NaCl and 10 mM NaHCO_3_, once every three days. Plant phenotypes were recorded at 15 and 20 days after stress initiation. After 20 days of stress treatment, leaves were collected from the plants for various physiological index assays.

### 4.2. Identification of A. thaliana Mutants

The SIGnAL website (http://signal.salk.edu/, accessed on 16 September 2021) was accessed to search for the mutant line IDs, gene identifiers, and primer sequences for mutant identification for each member of the PLC family in *A. thaliana*. The mutant line IDs and primer information for each gene are listed in Table S1. Upon obtaining the mutant seeds, they were sown in culture pots. When the mutant seedlings reached the four-leaf stage, leaves were collected, and genomic DNA was extracted using the genomic DNA extraction kit (CWBio, Taizhou, China). The homozygous mutants were identified using the three-primer PCR method.

### 4.3. Measurement of Physiological Indexes

#### 4.3.1. Measurement of Dry Weight, Fresh Weight and Water Content

Leaves of *A. thaliana* seedlings under the same growth conditions that had been subjected to stress for 20 days were selected. The surface moisture was wiped off, and the fresh weight of the seedlings was recorded. The seedlings were then placed in an oven at 105 °C for 30 min to inactivate enzymes, followed by drying at 70 °C for 2 days until constant weight was achieved. The dry weight was then recorded. The relative water content was calculated by subtracting the dry weight from the fresh weight, dividing the difference by the fresh weight, and expressing the result as a percentage.

#### 4.3.2. Measurement of Malondialdehyde, Proline and Chlorophyll Content

Three *A. thaliana* leaves were collected per sample, washed thoroughly, and blotted dry. For each biological replicate, 0.1 g of leaf tissue was weighed and ground into a homogenate with 2 mL of 10% trichloroacetic acid (TCA). The homogenate was then centrifuged at 12,000× *g* for 15 min at low temperature in a high-speed refrigerated centrifuge (Eppendorf, Hamburg, Germany). Subsequently, 1.5 mL of the clear supernatant was transferred to a new centrifuge tube. To this supernatant, 1.5 mL of 0.5% thiobarbituric acid (TBA) solution was added. The tube was then placed in a boiling water bath for 30 min to allow the reaction to proceed. After the reaction, the tube was rapidly cooled and centrifuged again at high speed. Finally, the absorbance of the resulting clear supernatant was measured at wavelengths of 532 nm, 450 nm, and 600 nm. The MDA content was then calculated based on the measured optical density (OD) values using the Nanjing Jiancheng Malondialdehyde (MDA) Assay Kit (Nanjing Jiancheng Bioengineering Institute, Nanjing, China).

The contents of chlorophyll, soluble sugar, and proline were determined using commercial assay kits following the manufacturers’ protocols. Specifically, the chlorophyll assay kit, plant soluble sugar content test kit, and proline assay kit (all procured from Nanjing Jiancheng Bioengineering Institute, Nanjing, China) were employed for the respective analyses.

#### 4.3.3. Measurement of Na^+^, K^+^ Concentrations and K^+^/Na^+^ Ratio

To determine the contents of sodium (Na^+^) and potassium (K^+^) ions, leaf samples were collected after 20 days of salt–alkali stress treatment. The leaf material was thoroughly rinsed with distilled water several times to remove any surface contaminants. Subsequently, the samples were oven-dried at 105 °C for 30 min to deactivate enzymes (killing green), and then further dried at 80 °C until a constant weight was achieved (approximately 48 h).

For ion extraction, 0.5 g of the dried sample was digested with 5 mL of sulfuric acid (H_2_SO_4_, density 1.84 g/mL) in a digestion tube. The digestion process was repeated three times to ensure complete breakdown of the plant material. After digestion, the resulting solution was diluted to a final volume of 100 mL with distilled water (acid was added to water with constant stirring to dissipate heat). An aliquot of 1 mL was then taken from this diluted solution and further diluted with 9 mL of distilled water. The concentrations of Na^+^ and K^+^ in the final solution were measured using a flame photometer (PerkinElmer, Waltham, MA, USA) according to the instrument’s standard protocol. The K^+^/Na^+^ ratio was subsequently calculated based on the obtained concentrations of the two ions. Three biological replicates were performed for each treatment to ensure data reliability.

#### 4.3.4. Histochemical Detection of H_2_O_2_ and O_2_^−^ Accumulation

The accumulation of hydrogen peroxide (H_2_O_2_) and superoxide anion (O_2_^−^) in leaves was detected histochemically using 3,3-diaminobenzidine (DAB) and nitroblue tetrazolium (NBT) staining, respectively, following established protocols with minor modifications.

For H_2_O_2_ detection, the DAB working solution was freshly prepared by mixing 900 µL of phosphate-buffered saline (PBS) with 50 µL of Solution A and 50 µL of Solution B (components of the DAB staining kit). Leaf samples were immersed in the DAB solution and incubated in the dark for 5 h at room temperature to allow staining. After incubation, the leaves were transferred into a destaining solution. The glass vessel containing the leaves and destaining solution was then heated in a boiling water bath until the leaves were completely bleached (chlorophyll removed). The destained leaves were subsequently washed twice with 95% ethanol to remove any residual destaining agent and photographed for documentation. The stained area, indicative of H_2_O_2_ accumulation, was quantified using ImageJ 1.53s software (National Institutes of Health, Bethesda, MD, USA) by calculating the percentage of brown staining area relative to the total leaf area.

For O_2_^−^ detection, the NBT staining solution was prepared by mixing 480 µL of 25× NBT with 1 mL of 1× AP reaction buffer, followed by the addition of 480 µL of 25× BCIP and thorough mixing. Leaf samples were then submerged in the NBT solution and incubated in the dark for 5 h at room temperature. Following staining, the leaves were subjected to the same destaining process as described above, using the destaining solution in a boiling water bath until fully bleached. The destained leaves were washed twice with 70% ethanol and photographed. The blue formazan precipitate, indicating O_2_^−^ accumulation, was also quantified by calculating the stained area percentage using ImageJ software. For each treatment, at least three biological replicates were analyzed to ensure the consistency of the results.

### 4.4. RNA Extraction and qRT-PCR

Plant tissue samples were ground into fine powder in liquid nitrogen. The tissue powder was then mixed with Trizol reagent at a ratio of 50–100 mg of tissue per 1 mL of Trizol. The homogenate was centrifuged at 2000× *g* for 5 min, and the supernatant was carefully transferred to a new tube. Chloroform was added at a volume of 200 μL per 1 mL of Trizol used. The mixture was inverted vigorously to mix and then allowed to stand until phase separation occurred. The sample was then centrifuged at 12,000× *g* for 15 min at 4 °C in a refrigerated high-speed centrifuge. The clear upper aqueous phase was carefully transferred to a new, labeled centrifuge tube. An equal volume of isopropanol was added to the recovered aqueous phase, mixed thoroughly, and allowed to stand until phase separation was observed. The mixture was centrifuged at 12,000× *g* for 10 min at 4 °C. The resulting RNA pellet was washed with 1 mL of 75% ethanol, gently pipetted to resuspend the pellet, and centrifuged again at 12,000× *g* for 5 min at 4 °C. The supernatant was carefully removed with a pipette. The RNA pellet was then dissolved in 30 μL of pre-aliquoted DEPC-treated water, and its concentration was determined.

The extracted RNA was reverse-transcribed using the PrimeScript™ 1st Strand cDNA Synthesis Kit (TAKARA, Kusatsu, Japan). The reverse transcription reaction was performed with 1 μg of RNA in a 10-μL reaction system. The resulting cDNA was diluted 50-fold and used as the template for quantitative real-time PCR (qRT-PCR). The gene sequences, gene-specific primers and internal reference primer sequences used for qRT-PCR are listed in Tables S2 and S3. The qRT-PCR was carried out using the One Step TB Green^®^ PrimeScript™ RT-PCR Kit II (TAKARA, Kusatsu, Japan) according to the manufacturer’s instructions. Each sample was analyzed in four replicates.

### 4.5. Stomatal Observation

For stomatal observation, three *A. thaliana* leaves were placed in a 10 mL centrifuge tube containing 6 mL ethanol and 1 mL glacial acetic acid (6:1, *v*/*v*) for fixation. The samples were fixed for 24 h, during which the tubes were inverted frequently to ensure thorough contact between the leaves and the fixation solution. Subsequently, the leaves were treated with 70% ethanol for 30 min, with the solution replaced several times until the leaves became completely decolorized and transparent. The transparent leaves were then transferred into a freshly prepared clearing solution containing chloral hydrate and glycerol, and incubated for an additional 6 h. Stomata on the leaf epidermis were then observed and photographed under a fluorescence inverted microscope (Leica, Wetzlar, Germany). Stomatal aperture area was measured using ImageJ software to evaluate stomatal changes under stress conditions.

### 4.6. DAB and NBT Staining

For histochemical detection of reactive oxygen species, DAB and NBT staining were performed according to a previously described method with minor modifications [52]. Briefly, for DAB staining, leaf samples were immersed in the DAB staining solution (900 μL PBS, 50 μL solution A, and 50 μL solution B) and incubated in the dark at 28 °C for 5 h. For NBT staining, leaves were incubated in the NBT working solution (480 μL 25× NBT and 480 μL 25× BCIP in 1 mL 1× AP reaction buffer) at 28 °C for 5 h in the dark. After staining, the leaves were transferred to a bleaching solution and heated in a boiling water bath until completely destained. The destained leaves were washed twice with 95% ethanol (for DAB) or 70% ethanol (for NBT) and photographed. The stained areas, representing H_2_O_2_ and superoxide anion accumulation, respectively, were quantified using ImageJ software. Three independent biological replicates were performed, and samples in each biological replicate were obtained from four different plants.

### 4.7. Leaf Temperature Measurement

Leaf surface temperature was measured after 5 days of salt–alkali stress. Wild-type and *atplc1* mutant seedlings were placed vertically at the same height and simultaneously captured using a Testo 885 thermal imaging camera (Testo SE & Co. KGaA, Titisee-Neustadt, Germany). The thermal camera was equipped with a temperature range of −30 to 100 °C and a thermal sensitivity of <0.10 °C. Images were taken from a distance of approximately 15 cm with an emissivity setting of 0.97. Average leaf temperatures were analyzed using Testo IRSoft V4.1 software.

### 4.8. Statistical Analysis

Quantitative data were expressed as mean ± standard deviation (SD). Independent-samples *t*-test was used for pairwise group comparisons. For the comparison of survival rates among multiple groups, Tukey’s honestly significant difference (HSD) post hoc test was employed following one-way ANOVA. Statistical significance was defined at *p* < 0.05, and *p* < 0.01 was regarded as highly significant.

## 5. Conclusions

In summary, *AtPLC1* plays a critical positive regulatory role in the *A. thaliana* response to salt–alkali stress. This gene functions by maintaining normal stomatal closure in response to stress signals, effectively limiting transpirational water loss and regulating leaf thermal balance. Concurrently, *AtPLC1* mitigates stress-induced oxidative damage through modulation of sodium/potassium ion homeostasis and activation of the antioxidant system, thereby ensuring plant survival under salt–alkali conditions. Its functional deficiency leads to impaired stomatal closure, excessive transpiration, exacerbated ROS burst, and disturbed ion balance, ultimately resulting in significantly reduced salt–alkali tolerance.

## Data Availability

The datasets generated and/or analyzed in the present study are available from the corresponding author on reasonable request.

## Supplementary Materials

The following supporting information can be downloaded at: https://www.mdpi.com/article/10.3390/plants15172633/s1, Figure S1: Genotypic identification of homozygous *atplcs* mutants by PCR; Table S1: Mutant information and primers for homozygous identification; Table S2: Primers used for expression analysis of PLC genes; Table S3: Protein and CDS sequences of AtPLC1 to AtPLC9.

## Abbreviations

The following abbreviations are used in this manuscript:

PLC	Phospholipase C
PI-PLC	Phosphatidylinositol-specific phospholipase C
NPC	Non-specific PLC
MDA	Malondialdehyde
IP_3_	1,4,5-trisphosphate
DAG	Diacylglycerol
PA	Phosphatidic acid
ROS	Reactive oxygen species
TCA	Trichloroacetic acid
TBA	Thiobarbituric acid
OD	Optical density
