# Supplementary material for: Loss of AtPLC1 Impairs Salt–Alkali Tolerance via Disruption of Stomatal Regulation and Redox Homeostasis in Arabidopsis thaliana
Source: Plants (Basel). 2026 Aug 28;15(17):2633. doi: 10.3390/plants15172633 (PMC13567353; doi:10.3390/plants15172633)
Supplement: Supplementary file 1 [file plants-15-02633-s001.zip › Figure S1.pdf]

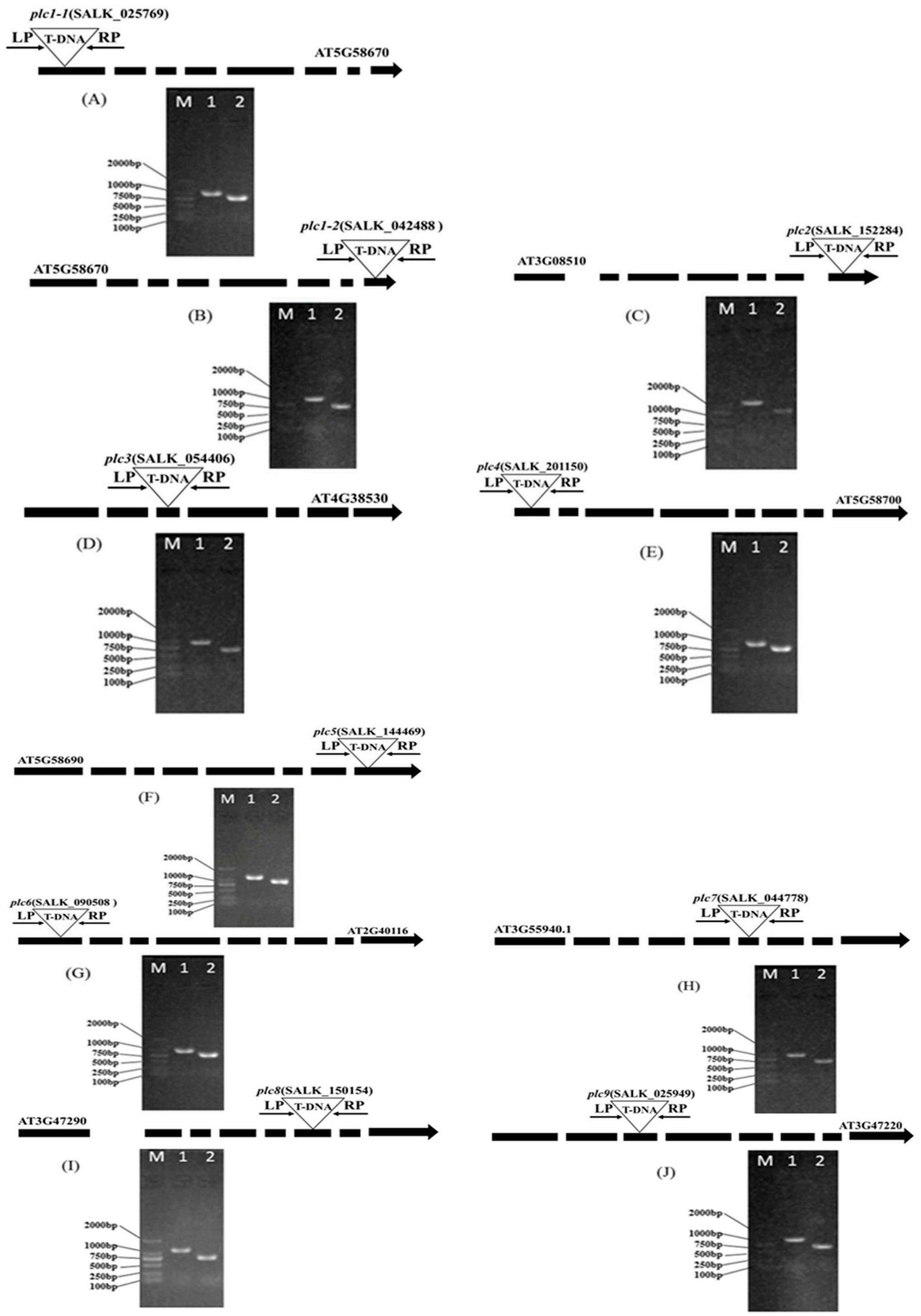

Figure S1. Genotypic identification of homozygous *atplcs* mutants by PCR. (A–J) Genotyping results for *atplc1-1* (A), *atplc1-2* (B), *atplc2* (C), *atplc3* (D), *atplc4* (E), *atplc5* (F), *atplc6* (G), *atplc7* (H), *atplc8* (I), and *atplc9* (J) mutants, respectively. Three primers were used in each PCR reaction: a left gene-specific primer (LP), a right gene-specific primer (RP), and a T-DNA left-border-specific primer (LB). The LP/RP primer pair amplifies the wild-type allele, while the LP/LB primer pair amplifies the mutant allele. M, DNA marker DL2000. Lane 1, wild-type (WT) control; lane 2, homozygous knockout mutant.
